# CLDN18.2-Targeted Therapy in Gastrointestinal Cancers

**DOI:** 10.3390/cancers17233764

**Published:** 2025-11-25

**Authors:** Andrea Dominguez Wiscovitch, Ricardo J. Sanchez Mendez, Jennifer Chuy

**Affiliations:** 1 Department of Medicine, NYU Langone Health, New York, NY 10016, USA; 2 NYU Grossman School of Medicine, NYU Langone Health, New York, NY 10016, USA; ricardo.sanchez-mendez@nyulangone.org; 3 Perlmutter Cancer Center, NYU Langone Health, New York, NY 10016, USA

**Keywords:** claudin18.2, gastric cancer, gastroesophageal junction adenocarcinoma, pancreatic cancer, biliary tract cancer

## Abstract

Gastrointestinal cancers, including gastric, gastroesophageal junction, pancreatic, and biliary tract cancers, remain associated with poor outcomes due to late diagnosis and limited effective treatment options. Claudin-18.2 (CLDN18.2), a tight junction protein primarily found in the gastric epithelium and ectopically expressed in gastrointestinal tumors, has emerged as a promising therapeutic target across these diseases. This narrative review expands on existing discussions surrounding CLDN18.2-directed therapy in gastric and gastroesophageal cancer and provides a comprehensive, updated analysis of the rapidly evolving therapeutic landscape across multiple gastrointestinal malignancies, including pancreatic and biliary tract cancers. We summarize key developments following the approval of the monoclonal antibody zolbetuximab and critically evaluate emerging modalities, including bispecific antibodies, antibody–drug conjugates, and chimeric antigen receptor T-cell therapies, highlighting differences in mechanisms of action, efficacy, toxicity profiles, and mitigation strategies. We also discuss the clinical relevance of CLDN18.2 and PD-L1 co-expression, the rationale for pairing CLDN18.2-targeted therapy with immune checkpoint inhibitors, and early data supporting combination approaches. Additionally, we examine tumor heterogeneity, biomarker challenges, and emerging resistance mechanisms, alongside strategies to overcome them. Finally, we identify current limitations in the field, including inconsistent CLDN18.2 testing criteria, and outline prioritized future directions to optimize integration of CLDN18.2-directed therapies across gastrointestinal cancers. By looking beyond zolbetuximab and incorporating cross-platform comparison, immuno-oncology considerations, and multi-tumor context, this review provides a broad and forward-looking framework to guide clinical application and next-generation research in CLDN18.2-targeted therapy.

## Simple Summary

Gastrointestinal cancers such as gastroesophageal, pancreatic, and biliary tract cancers are often diagnosed late and remain difficult to treat, with limited benefit from standard therapies. Claudin-18.2 (CLDN18.2), a protein normally found in the stomach lining, is abnormally expressed in several gastrointestinal cancers and has emerged as a promising therapeutic target. Zolbetuximab, the first CLDN18.2-directed therapy, has improved survival in advanced gastric cancer, leading to growing interest in this pathway. This review provides a comprehensive overview of CLDN18.2-targeted therapies, moving beyond zolbetuximab to highlight next-generation approaches, such as bispecific antibodies, antibody–drug conjugates, and cellular therapies. We also discuss CLDN18.2 expression in pancreatic and biliary tract cancers, its relationship with PD-L1, and potential synergy with immunotherapy in gastric cancers, as well as emerging resistance mechanisms and strategies to overcome them. Finally, we outline key future priorities to advance CLDN18.2-directed treatment across gastrointestinal cancers.

## 1. Introduction

Gastrointestinal (GI) cancers, including gastric cancer (GC), gastroesophageal junction cancer (GEJC), pancreatic cancer (PC), and biliary tract cancers (BTCs), represent a significant global health burden. GC is the fifth-most commonly diagnosed cancer and the third leading cause of cancer-related mortality globally, with close to 1 million new cases and approximately 700,000 deaths in 2022 [1]. Incidence rates are particularly high in East Asia, Eastern Europe, and South America [1]. There were an estimated 1.1 million deaths related to both gastric and esophageal cancers globally in 2022 [1]. PC, while less common, is one of the deadliest malignancies, with a five-year survival rate of approximately 13% [2]. BTCs, encompassing intrahepatic (iCCA) and extrahepatic cholangiocarcinoma (eCCA) as well as gallbladder cancer (GBC), are relatively rare but highly aggressive, often diagnosed at advanced stages with limited treatment options and a poor prognosis [3]. While systemic therapy with chemotherapy, targeted therapy, and immunotherapy remain the mainstay of treatment for advanced disease, their survival outcomes are modest, emphasizing the need for novel therapies that address the complex molecular landscapes of these diseases.

The shift toward personalized medicine with the introduction of targeted therapies has revolutionized cancer treatment, offering hope for more effective and less toxic interventions. These therapies are designed to target tumor-specific molecular alterations, thereby sparing healthy tissue and minimizing side effects. One of the emerging molecular targets is claudin-18.2 (CLDN18.2), a tight junction protein that is predominantly expressed in the normal gastric epithelium but becomes aberrantly expressed in different GI malignancies, including GC/GEJC, PC, and BTCs [4,5,6]. This altered expression profile has made CLDN18.2 an attractive therapeutic target.

Recent clinical advances have highlighted the potential of CLDN18.2-targeted therapies. Zolbetuximab (Vyloy), an anti-CLDN18.2 monoclonal antibody (mAb), was recently approved by the U.S. FDA for use in combination with chemotherapy in patients with HER2-negative, CLDN18.2-positive advanced GC/GEJC [7]. The pivotal phase III SPOTLIGHT and GLOW trials demonstrated significant improvements in progression-free survival (PFS) and overall survival (OS) with this combination compared to chemotherapy alone [8,9]. These findings have not only validated CLDN18.2 as a clinically actionable biomarker in GC/GEJC but have also led to the development of numerous anti-CLDN18.2 agents under investigation and prompted exploration into CLDN18.2’s role across other malignancies, such as PC and BTCs. In this narrative review, we extend beyond zolbetuximab to provide a comprehensive and forward-looking evaluation of CLDN18.2-directed therapies across GI cancers, including next-generation platforms (bispecific antibodies [BsAbs], antibody–drug conjugates [ADCs], and chimeric antigen receptor [CAR] T-cell therapies), comparative safety and mitigation strategies, the role of CLDN18.2/PD-L1 co-expression and immunotherapy combinations, emerging resistance mechanisms, and prioritized future research directions.

## 2. Background on CLDN18.2

### 2.1. CLDN18.2 Structure and Function

Tight junctions are a collection of proteins that join epithelial or endothelial cells together to form a paracellular barrier. This seal prevents passage of water or solutes through the spaces between cells and can be selectively permeable for certain molecules depending on a cell’s specific function [10]. Claudins are a family of at least 27 transmembrane proteins that are expressed in a variety of tissues and form an integral part of tight junctions. Claudin-18 (CLDN18) is one of the main components of tight junctions found in stomach and lung epithelial cells. CLDN18 is composed of two extracellular loops, four transmembrane domains, and a cytoplasmic domain. Both the C-terminus and N-terminus are in the cytoplasm [11]. The *CLDN18* gene on chromosome 3q22 generates two splice variants, CLDN18.1 and CLDN18.2, which are predominantly found in lung and gastric tissue, respectively [4,12].

CLDN18.1 is primarily expressed in alveolar type I epithelial cells and is important in the formation of the alveolar epithelial barrier and architecture. One study of mice with CLDN18 knockout found that absent CLDN18 expression resulted in impaired alveolarization and postnatal lung injury [13].

**Figure 1:**
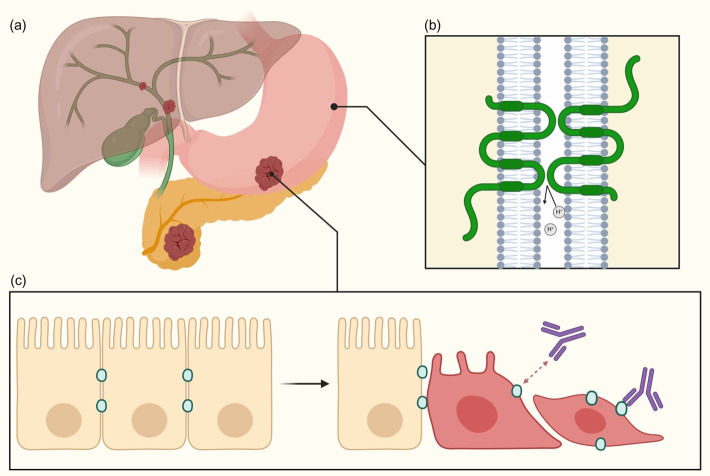
Structure and function of CLDN18.2. (**a**) The therapeutically relevant isoform CLDN18.2 is found in healthy gastric mucosa and is also expressed in a variety of tumors, including GC/GEJC, PC, and BTC. (**b**) It is a transmembrane tight junction protein that mediates paracellular H+ ion in gastric tissue. In healthy epithelium, CLDN18.2 is confined to the intercellular membrane, rendering it inaccessible to antibody binding. (**c**) During GC malignant transformation, changes in cell polarity expose CLDN18.2 on the tumor cell surface, allowing recognition by therapeutic mAbs. Created in BioRender. Sanchez-Mendez, R. (2025) https://BioRender.com/rtkxwu6, accessed on 16 September 2025.

CLDN18.2 is preferentially expressed in healthy gastric mucosa and has become a molecular target of interest in the treatment of GC [4]. It plays a crucial role in the barrier and selective permeability of the tight junctions in normal gastric epithelial cells by controlling the passage of ions such as Na+ and H+ [14,15] (Figure 1). The importance of this function was highlighted by studies showing that mice with CLDN18 knockdown developed severe gastritis within days, indicating that CLDN18.2 helps prevent paracellular leakage of H+ ions, a key protective function [14,15]. Therefore, it directly impacts the acidic environment of the stomach, and dysfunction or changes in expression may contribute to poor acidity regulation.

### 2.2. CLDN18.2 Expression and Role in Gastrointestinal Tumors

#### 2.2.1. CLDN18.2 Expression in Gastric and Gastroesophageal Junction Cancer

Some studies in GC have shown that there is a downregulation of CLDN18.2 when compared to normal gastric tissue [4,16,17]. At a population level, transcriptional analyses involving the public Cancer Genome Atlas Program database have found that CLDN18 is downregulated in GC compared to normal gastric tissue, though it is still highly expressed compared to other cancers [17,18]. However, despite the downregulation, CLDN18.2 expression remains stable throughout the malignant transformation process and is readily identified in primary and metastatic sites [19,20,21].

In studies defining CLDN18.2 positivity as ≥75% of tumor cells expressing moderate-to-high membranous CLDN18.2 staining on immunohistochemistry (IHC), such as the SPOTLIGHT and GLOW trials, it has been found that approximately 24–38% of GC/GEJCs are CLDN18.2-positive [8,9,22]. Previous studies have used lower positivity cutoffs, so reported expression rates have varied depending on the criteria used [23].

While the expression of CLDN18.2 in GC has been well-studied, its exact role in oncogenesis remains unclear. One study found that CLDN18 knockout mice were more likely to develop intraepithelial neoplasm and polypoid tumors, suggesting that it may play a tumor-suppressive role [24]. Another in vitro study on GC cells identified that in a tumor microenvironment (TME), CLDN18.2 directly interacted with cancer-associated fibroblasts (CAFs) to mediate adhesion between malignant cells, contributing to invasion and metastatic spread [25]. Of note, other studies have explored the *CLDN18-ARHGAP26* fusion gene, which is associated with high expression of CLDN18.2. It has been postulated that this fusion destroys the structure of wild-type CLDN18.2 and contributes to GC progression and invasion through the breakdown of the intercellular barrier and cell–cell adhesion [26]. Therefore, while CLDN18.2 appears to serve as a tumor suppressor in physiological conditions, its dysregulation and complex relationship with the TME suggests it may contribute to tumor progression.

Although a direct causal link between CLDN18.2 and carcinogenesis remains to be elucidated, its aberrant expression and effect on cellular polarity may still contribute to tumor progression and invasion, making it an ideal molecular target [27]. Normally, CLDN18.2 epitopes are present in the tight junction complex in normal gastric mucosa, making the protein inaccessible for antibody binding. However, during malignant transformation and the epithelial-to-mesenchymal transition common in malignancy, alterations in polarity and expression lead to the epitope becoming exposed on the tumor cell surface [27,28] (Figure 1). This stable expression across disease stages, coupled with CLDN18.2 epitope’s exposure on the tumor cell surface, has made CLDN18.2 an attractive and accessible target for novel therapeutic agents. By serving as a marker that is preferentially targetable by mAbs, it may be uniquely useful in reaching malignant cells while minimizing effects on healthy tissue.

Recent research has attempted to directly analyze the predictive and prognostic impact of CLDN18.2 expression. Overall, no clear associations have been found. A 2021 meta-analysis examined CLDN18.2 testing as a prognostic tool in GC with either any level of tumor cell 2+ to 3+ staining or a cutoff of ≥40% cell staining on IHC [29]. They also found that there was no statistically significant association between CLDN18.2 positivity and TNM stage, histologic grade, or Lauren histologic subtype classification.

#### 2.2.2. CLDN18.2 Expression in Pancreatic Cancer

Besides its normal expression in gastric epithelium, CLDN18.2 expression is also upregulated or altered in cancerous tissues, including in esophageal, PC, and BTCs, where it may function as a tumor promoter [4,6,17,30].

CLDN18.2 expression has been found in PC, particularly in pancreatic ductal adenocarcinoma (PDAC), as well as in precancerous lesions such as pancreatic intraepithelial neoplasias (PanINs), intraductal papillary mucinous neoplasms (IPMNs), and mucinous cystic neoplasms (MCNs). One recent study utilized publicly available genomics data from the Cancer Genome Atlas Program to confirm that CLDN18.2 was highly expressed in PC along with GC/GEJC [17,18]. Interestingly, there is minimal-to-no expression of CLDN18.2 in nonmalignant pancreatic tissue, so its expression in PDAC and its precursor lesions has been suggested to indicate that CLDN18.2 could be a key factor in the early stages of PDAC development [31,32,33].

To date, two studies have assessed CLDN18.2 expression in PDAC using the same immunohistochemical CLDN18.2 positivity criteria used in the hallmark zolbetuximab trials, SPOTLIGHT and GLOW (≥75% tumor cells with ≥2+ membranous staining). These studies showed that around 30.4–32.5% of PDACs are positive for CLDN18.2, suggesting that around one-third of patients could be eligible for zolbetuximab therapy using the same inclusion criteria [34,35].

#### 2.2.3. CLDN18.2 Expression in Biliary Tract Cancer

Similar to PC, CLDN18.2 expression has been noted in BTCs. Shinozaki et al. introduced the potential therapeutic relevance of CLDN18 in BTCs, reporting its expression in 23–82% of BTCs, defining positivity as ≥2+ membranous staining in ≥25% of tumor cells in IHC [6]. In a recent study looking at CLDN18 expression in BTCs, Angerilli et al. reported CLDN18 expression in 29.5% (70/237) of BTC samples analyzed. When using a CLDN18 positivity definition of moderate-to-strong membranous staining in ≥75% of tumor cells, as used in the SPOTLIGHT and GLOW trials, it was observed in 5.5% (13/237) of BTCs [36]. Expression and positivity rates varied significantly across BTC subtypes, with higher prevalence in GBCs (62.5% expression, 14.6% positivity) and eCCAs (53.4% expression, 8.6% positivity) compared to iCCAs (12.9% expression, 2.0% positivity) [36]. These findings are clinically significant because GBCs and eCCAs have fewer therapeutic options compared to iCCAs [36]. Comparisons with previous studies, such as Kinzler et al., support these findings but also highlight variability in detection methods [36,37].

Similar to PDAC, CLDN18.2 seems to serve as a tumor promoter in BTCs despite not being expressed in healthy tissue [36]. For example, one study identified that constitutive activation of the EGFR, ERK1/2, and PKC pathways induced ectopic overexpression of CLDN18 and resulted in bile duct tumorigenesis in mice [38]. Though they did not distinguish between the two CLDN18 isoforms, this suggests that CLDN18.2 is a promising target for advanced BTCs.

## 3. Testing CLDN18.2 Expression and Tumoral Heterogeneity

IHC is the main method that has been used to determine CLDN18.2 expression in clinical trials and in clinical practice. It enables direct visualization of CLDN18.2 expression in tumor tissues and plays a crucial role in determining eligibility for therapies targeting CLDN18.2 [39]. The VENTANA CLDN18 (43-14A) RxDx Assay has been the most widely used for detecting CLDN18.2 expression in tumors in major trials and it was approved as a companion diagnostic device by the FDA [7].

However, it is important to note inconsistencies in determining what qualifies as a positive expression, given differences among laboratories and the absence of a universal cutoff value in various studies. For example, the FAST trial defined ≥ 40% of tumor cells with moderate-to-high (+2 to +3 on IHC) CLDN18.2 expression as the cutoff for positivity [23]. However, in their subgroup analysis using a cutoff of ≥70%, a benefit in PFS and OS was noted compared to the lower expression ranges. This suggested that CLDN18.2-targeted therapy may preferentially benefit high-expressing tumors, so later trials such as SPOTLIGHT and GLOW used ≥75% of tumor cells with moderate-to-high expression as the cutoff value [8,9,23]. There remains a wide variability in tumor expression standards, and to our knowledge, no studies have employed quantitative insights such as receiver operating characteristic (ROC) analysis to elucidate the optimal percentage of tumor cells positivity cutoff on IHC. Another method that has been utilized to determine CLDN18.2 expression is gene expression analysis, such as quantitative reverse transcription-polymerase chain reaction, although it is less commonly used in clinical practice [40]. This method is limited by its inability to account for intratumoral heterogeneity, which is more adequately captured by IHC. One study investigated detecting CLDN18.2 in circulating tumor cells with good concordance to IHC, though the sample size was small and more evidence is needed to assess viability [41]. Overall, commercially available IHC assays remain the gold standard for evaluating CLDN18.2 expression at this time.

Since eligibility for CLDN18.2-directed treatment is tied to quantitative tumor expression, adequate sample selection and testing methodology are important for guiding treatment options for patients. CLDN18.2 expression has been found to show intratumoral variability in 33–40% of GCs [20,42]. This parallels to the well-characterized heterogeneity of HER2 expression, where sampling variability has historically influenced therapeutic selection and outcomes. One study assessing CLDN18.2 found similar results between individual GC biopsy cores, where 61.3% of patients showed different CLDN18.2 expression results between tumor microarray cores [43]. However, comparisons between whole tissue sections did not identify this discrepancy in expression, highlighting the limitations of small-core sampling approaches. Similarly, intratumoral heterogeneity in CLDN18.2 expression has been noted in PC [44,45] and BTC samples [36], although data remain limited and require confirmation in larger studies.

Intertumoral heterogeneity also contributes to diagnostic complexity. Discordance in CLDN18.2 positivity has also been found between primary sites and metastases in 20–25% of GCs [46,47], reflecting patterns seen with HER2 expression. For instance, in the GASTHER-1 study, up to 8% of patients with HER2-negative primary GCs on endoscopic biopsy were subsequently found to be HER2-positive on follow-up metastatic site biopsies [48].

Tumor heterogeneity may have clinical implications by affecting both treatment efficacy and diagnostic management. HER2 intralesional heterogeneity has been found to predict poorer responses to trastuzumab [49], raising the possibility that heterogeneous CLDN18.2 expression may similarly limit the efficacy of zolbetuximab and other CLDN18.2-targeted therapies. As such, clinicians may need to rely on multiple endoscopic biopsies from primary GC sites to improve diagnostic accuracy, with some suggesting that six biopsies may be optimal [42,47].

## 4. CLDN18.2-Targeted Therapy

**Figure 2:**
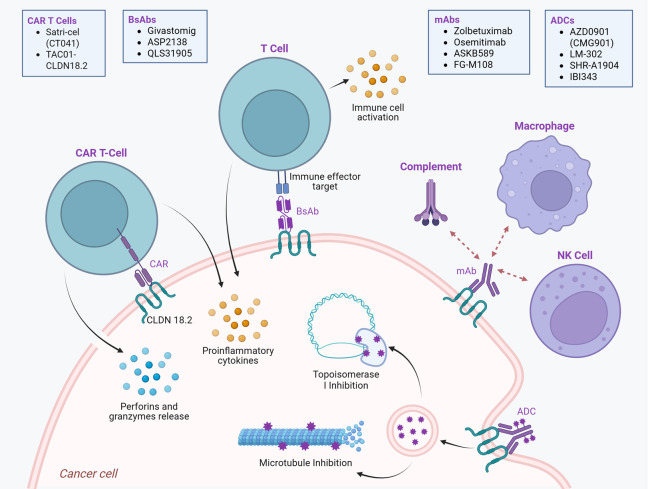
Mechanisms of action of CLDN18.2-directed therapies. CLDN18.2-directed therapeutic strategies include mAbs, ADCs, BsAbs, and CAR T-cell therapies. mAbs (e.g., zolbetuximab) directly bind to CLDN18.2 and induce antibody- and complement-dependent cytotoxicity. ADCs deliver cytotoxic payloads such as microtubule or topoisomerase inhibitors after internalization. BsAbs engage T-cells and other immune effectors via concurrent binding of CLDN18.2 and costimulators such as CD3 or 4-1BB. CAR T-cells are genetically engineered to recognize CLDN18.2 and directly release perforins, granzymes, and cytokines to lyse tumor cells. All of these modalities harness CLDN18.2’s selective tumor expression to achieve targeted cytotoxicity or immune activation in GI malignancies. Created in BioRender. Sanchez-Mendez, R. (2025) https://BioRender.com/elpo01j, accessed on 16 September 2025.

With CLDN18.2’s restricted expression pattern in normal gastric epithelium tissue and its aberrant expression in several GI cancers, CLDN18.2 is an ideal target for developing novel therapies. Various therapeutic modalities made to target CLDN18.2, including mAbs, ADCs, BsAbs, and CAR T-cell therapies, are currently under investigation in clinical trials. Figure 2 demonstrates the different therapeutic approaches for targeting CLDN18.2. This section reviews the latest developments in CLDN18.2-targeted therapies, highlighting a few compounds in each category along with key findings from their clinical trials.

### 4.1. Monoclonal Antibodies (mAbs)

#### 4.1.1. Zolbetuximab (IMAB362/ClaudiXimab/Vyloy)

Zolbetuximab is a humanized mAb directed against CLDN18.2 and represents the first approved therapy for CLDN18.2-positive GI cancers. It received FDA approval in combination with chemotherapy as a first-line treatment for patients with unresectable, locally advanced, or metastatic, HER2-negative, CLDN18.2-positive GC/GEJC [7]. The antibody recognizes a specific epitope located within the first extracellular loop of CLDN18.2, enabling selective tumor targeting with strong binding affinity [29]. Zolbetuximab’s anti-tumor activity is mediated primarily through antibody-dependent cellular cytotoxicity (ADCC) and complement-dependent cytotoxicity (CDC) in CLDN18.2-positive GC/GEJC cell lines, as demonstrated in preclinical studies [8,27].

The MONO trial was a phase II trial that assessed zolbetuximab monotherapy in 54 patients with recurrent or refractory advanced GC or lower esophageal adenocarcinoma (EAC) exhibiting moderate-to-strong CLDN18.2 expression in ≥50% of tumor cells. Across 43 assessable patients, the objective response rate (ORR) was 9%, with a clinical benefit rate (CBR) of 23%. A subgroup analysis revealed that patients with ≥70% tumor cells expressing CLDN18.2 had a higher ORR of 14%. Zolbetuximab showed moderate clinical activity with an overall tolerable safety profile. Treatment-related adverse events (TRAEs) occurred in 82% of patients, with the most common being nausea (61%), vomiting (50%), and fatigue (22%) [50].

The ILUSTRO phase II study, which defined CLDN18.2 positivity as ≥50% of tumor cells demonstrating moderate-to-strong staining, is composed of four cohorts. Zolbetuximab was evaluated as monotherapy in the third-line setting and in combination with chemotherapy (mFOLFOX6) in the first-line setting or with immunotherapy (pembrolizumab) in the third-line setting for advanced CLDN18.2-positive GC. Notably, ORR was 71.4% in the zolbetuximab plus chemotherapy arm, showing robust activity in the first-line setting [51]. While zolbetuximab with immunotherapy in the third-line setting showed minimal activity, this cohort comprised only three patients and thus limited conclusions can be made regarding the combination of this agent and immune checkpoint inhibitors (ICIs). Cohort 4 is evaluating zolbetuximab in combination with FOLFOX and nivolumab as first-line treatment in patients with HER2-negative, metastatic GC/GEJC with intermediate-to-high CLDN18.2 expression. No results available as of July 2025.

The FAST trial, another phase II study, compared zolbetuximab plus chemotherapy (EOX) versus chemotherapy alone as a first-line treatment for advanced CLDN18.2-positive GC/GEJC. CLDN18.2 positivity was defined as moderate-to-strong expression of CLDN18.2 in ≥40% tumor cells. PFS in the zolbetuximab plus EOX arm was significantly improved (7.5 months vs. 5.3 months) with a median OS of 13.0 months compared to 8.3 months in the chemotherapy-alone group [23]. This trial confirmed the value of adding zolbetuximab to chemotherapy in CLDN18.2-positive advanced GC. Notably, in patients with CLDN18.2 expression in ≥70% tumor cells, significant improvements in PFS and OS were observed, whereas in those with CLDN18.2 expression in 40–69% of tumor cells, PFS and OS were not significantly different. These results led to changes in positivity cutoff criteria in the following phase III trials SPOTLIGHT and GLOW to ≥75% cells.

The GLOW phase III study enrolled 507 patients with HER2-negative, locally advanced or metastatic GC/GEJC, randomized to receive either zolbetuximab plus CAPOX, or CAPOX plus placebo. The addition of zolbetuximab improved PFS (8.21 months vs. 6.80 months) and OS (14.39 months vs. 12.16 months) in the first-line setting. However, ORR between the two arms remained similar, highlighting that zolbetuximab’s benefit lies more in disease stabilization rather than tumor shrinkage [9].

The SPOTLIGHT phase III trial mirrored the GLOW study but used FOLFOX as the chemotherapy backbone. Zolbetuximab in combination with FOLFOX significantly improved PFS (10.6 months vs. 8.6 months) and OS (18.2 months vs. 15.5 months). Like GLOW, ORR did not differ significantly, suggesting again that the combination stabilizes the disease without inducing substantial tumor shrinkage [8].

A phase II trial assessing the efficacy of zolbetuximab plus chemotherapy as first-line therapy for patients with CLDN18.2-positive metastatic PC is ongoing, with estimated completion by August 2026 (NCT03816163).

Moreover, a phase III trial combining chemotherapy (CAPOX or mFOLFOX6) and pembrolizumab with or without zolbetuximab as first-line therapy in advanced or metastatic GC/GEJC whose tumors are HER2-negative, CLDN18.2-positive, and PD-L1-positive is underway (NCT06901531). This is a pivotal step in addressing the question of what is the preferred first-line treatment for patients co-expressing CLDN18.2 and PD-L1. Details about these studies and clinical trials for other mAbs in development can be found in Table 1.

#### 4.1.2. Osemitamab (TST001)

Osemitamab, formerly TST001, is a mAb engineered to improve CLDN18.2 binding and enhance ADCC activity through reduced fucosylation of the Fc region. Early-phase clinical trials suggest anti-tumor activity, including in tumors with low-to-moderate CLDN18.2 expression levels [58,59]. Preclinical studies also indicate that osemitamab exposure can increase PD-L1 expression on CLDN18.2-positive tumor cells [60].

The TranStar101 trial (NCT04396821) is a phase I/II study evaluating osemitamab as a single agent and in combination with chemotherapy and nivolumab in patients with GC/GEJC. Early data indicates osemitamab is generally well tolerated in this population [61]. The most frequently observed TRAEs were nausea (73.5%), vomiting (48.5%), and fatigue (30.9%). TRAEs of grade ≥ 3 that occurred in ≥3% of patients included nausea, vomiting, hypoalbuminemia, hypertension, infusion-related reaction, and increase in lymphocyte count across all dose levels [61].

The TranStar102 trial (NCT04495296) is another phase I/II study assessing osemitamab either alone or in combination with nivolumab and chemotherapy, or with nivolumab alone, in patients with advanced or metastatic GC/GEJC. Cohort C evaluates osemitamab plus CAPOX as first-line therapy. Positive CLDN18.2 expression was defined as ≥1+ membranous staining intensity in ≥10% of tumor cells. As of September 2023, 28 out of 42 patients achieved partial response (PR), of which 23 (54.8%) had been confirmed. The median duration of response (DoR) of the confirmed responses was 12.7 months (95% CI: 8.3-NE). For the 49 patients in the dose-expansion group, the estimated PFS was 14 months (95% CI: 5.9-NE) [62]. The most commonly reported TRAEs included nausea (65.4%), hypoalbuminemia (65.4%), and vomiting (46.2%), with most of them being grade 1 or 2 in severity [52].

Cohort G of the TranStar102 trial was designed to evaluate osemitamab in combination with CAPOX and nivolumab as first-line treatment for patients with HER2-negative or unknown advanced or metastatic GC/GEJC, regardless of CLDN18.2 and PD-L1 status. As of January 2025, 82 patients received osemitamab with CAPOX and nivolumab. In this cohort, 44 out of 82 patients achieved a confirmed PR and the ORR was 55.7%. Notably, patients with medium-to-high CLDN18.2 expression (≥2+ membranous staining in ≥40% of tumor cells) and known combined positive score (CPS) demonstrated enhanced clinical benefit. Among this subgroup (n = 26), the confirmed ORR was 68%, with a median PFS of 16.6 months (95% CI: 5.8–21.7). At data cutoff, 33 patients remained in survival follow-up, including 12 still receiving treatment. The median OS for the full cohort was 20.4 months (95% CI: 15.0-NE) [53]. All patients experienced TRAEs, with the safety profile being similar to cohort C [52,53,62]. Overall results suggest that the combination of TST001 plus nivolumab and CAPOX for the first-line treatment of patients with GC/GEJC is safe and well tolerated, with encouraging durable PFS and OS [53].

A phase III trial combining osemitamab, nivolumab, and chemotherapy as first-line therapy in CLDN18.2-positive advanced or metastatic GC/GEJC is planned, with estimated completion by January 2026 (NCT06093425).

#### 4.1.3. ASKB589

ASKB589 is a humanized anti-CLDN18.2 IgG1 mAb with high affinity and enhanced ADCC activity. In a phase I/II study, ASKB589 demonstrated a tolerable safety profile and encouraging anti-tumor activity as monotherapy in pretreated patients with CLDN18.2-positive advanced solid tumors, as well as in combination with CAPOX as first-line treatment for patients with CLDN18.2-positive advanced GC/GEJC (NCT04632108). ASKB589 as monotherapy showed an ORR of 22% and a disease control rate (DCR) of 89%. In the combination therapy subgroup, the ORR was 75% and the DCR was 100% [63]. The most common TRAEs included nausea, vomiting, and hypoalbuminemia [63]. Most recently, results have become available for cohort 5 of this trial, which evaluated ASKB589 in combination with chemotherapy as second-line treatment for advanced GC/GEJC [54]. As of December 2024, a total of 47 patients were enrolled in this cohort and treated with ASKB589 plus chemotherapy, primarily paclitaxel (n = 44). Of the 38 evaluable patients with moderate-to-high CLDN18.2 expression (≥40% tumor cells with ≥2+ intensity), the ORR was 34.2%, and the DCR was 71.1%. Specifically, for the ASKB589 plus paclitaxel subgroup (n = 35), the ORR was 31.1% and the DCR was 71.4%. In terms of survival outcomes, the median PFS in the intention-to-treat analysis was 5.26 months (95% CI: 2.66–7.06), and the median OS was 11.14 months (95% CI: 8.80–18.20). For the ASKB589 plus paclitaxel subgroup, the median PFS was 4.63 months (95% CI: 2.04–7.06) and median OS was 13.73 months (95% CI: 8.80–18.20). TRAEs were reported in all patients, with 26 patients (55.3%) experiencing grade ≥ 3 TRAEs. The most frequent grade ≥ 3 events included neutrophil count decrease (42.6%), WBC count decrease (17%), lymphocyte count decrease (8.5%), and hypoalbuminemia (8.5%). These findings indicate that ASKB589 plus chemotherapy, particularly with paclitaxel, demonstrates encouraging efficacy and manageable toxicity in the second-line setting for patients with CLDN18.2-positive GC/GEJC. Continued investigation in larger studies is warranted to further define its role.

Another phase I/II study evaluated ASKB589 combined with chemotherapy (CAPOX) and sintilimab (a PD-1 inhibitor) as first-line treatment for locally advanced, relapsed, and metastatic CLDN18.2-positive GC/GEJC, regardless of PD-L1 status (NCT05632939). This combination was found to have a tolerable safety profile as well as significant anti-tumor activity. A total of 40 out of 49 (81.6%) evaluable patients with medium-to-high CLDN18.2 expression (≥2+ membrane staining intensity in ≥40% tumor cells) achieved a partial or complete response, with a confirmed ORR of 73.5% (95% CI: 58.9–85.1) and a median DoR of 11.14 months (95% CI: 6.93-NE). All 49 patients achieved SD or better, with a DCR of 100% [55]. The preliminary data from all treated patients (n = 50) showed a PFS rate at nine months of 58.1% (95% CI: 42.2–71.0) and an OS rate at twelve months of 77.1% (95% CI: 61.2–87.2). In terms of safety, the combination of ASKB589 with chemotherapy and sintilimab showed similar results to previous studies with ASKB589 combined with chemotherapy, with side effects such as hypoalbuminemia, nausea, and vomiting [55].

Moreover, there is an ongoing phase III trial investigating ASKB589 in combination with CAPOX plus tislelizumab (anti-PD-1 antibody) as first-line therapy for patients with CLDN18.2-positive advanced stage GC/GEJC (NCT06206733).

#### 4.1.4. FG-M108 (M108)

FG-M108 or M108 is a second generation anti-CLDN18.2 mAb with enhanced target affinity and ADCC action. A phase I/II trial is evaluating FG-M108 plus CAPOX in the first-line setting for patients with CLDN18.2-positive advanced GC (NCT04894825). Cohort A received 300 mg/m^2^ dose of FG-M108 in combination with CAPOX, and cohort B received 600 mg/m^2^ dose of FG-M108 in combination with CAPOX [56]. In the most recently updated results, patients in cohort A with moderate-to-high CLDN18.2 expression (IHC 2+/3+ on ≥40% of tumor cells) had a confirmed ORR of 78% and a median PFS of 11.0 months (95% CI: 6.9–12.7), which were significantly better than those in cohort B [56]. Grade ≥ 3 TRAEs were observed in 37% of patients in cohort A and 18% of those in cohort B [56]. Overall, FG-M108 plus CAPOX showed a reasonable safety profile combined with promising anti-tumor activity in GC.

In light of these encouraging results, a phase III trial is underway to confirm the efficacy of FG-M108 plus CAPOX as first-line treatment for locally advanced unresectable or metastatic GC/GEJC with moderate-to-high CLDN18.2 expression (NCT06177041).

Other cohorts (C2 and D2) from the same phase I/II trial above (NCT04894825) are investigating FG-M108 plus gemcitabine and nab-paclitaxel in the first-line setting for patients with CLDN18.2-positive locally advanced or metastatic PC. Cohort C2 received 300 mg/m^2^ dose of FG-M108 in combination with gemcitabine and nab-paclitaxel, and cohort D2 received 600 mg/m^2^ dose of FG-M108 in combination with gemcitabine and nab-paclitaxel. The most updated results for this study show that among the 50 enrolled patients (39 patients in cohort C2, 11 patients in cohort D2), 47 (94%) had moderate-to-high CLDN18.2 expression [57]. A total of 44 patients out of 50 had at least one tumor assessment after baseline and were included in the efficacy analysis. For the 32 patients with moderate-to-high CLDN18.2 expression, the median follow-up time was 9.5 months (95% CI: 6.8–11.2), with a maximum treatment duration of 13 months. Confirmed ORR was 53.1% (95% CI: 34.7–70.9) and the DCR was 100% (95% CI: 89.1–100.0) [57]. Median PFS and DoR both reached 9.9 months, while median OS was 17.4 months, although OS data remain immature with 23 patients still alive at the time of analysis [57]. The treatment regimen was generally well tolerated. Treatment-emergent adverse events (TEAEs) occurred in all 32 patients, with 46.9% experiencing grade ≥ 3 events. The most common adverse events in cohort C2 and D2 were nausea (56.4% vs. 36.4%), vomiting (48.7% vs. 45.5%), and hypoalbuminemia (46.2% vs. 54.5%) [57]. These findings highlight the promising therapeutic potential of FG-M108 in combination with chemotherapy as first-line treatment for patients with CLDN18.2-positive PC, especially those with moderate-to-high CLDN18.2 expression.

### 4.2. Antibody–Drug Conjugates (ADCs)

ADCs combine mAbs with potent cytotoxic drugs, allowing for targeted delivery to tumor cells with the potential for reduced systemic toxicity. ADCs targeting CLDN18.2 aim to selectively deliver chemotherapy to tumors, enhancing efficacy and limiting off-target effects.

#### 4.2.1. AZD0901 (CMG901)

AZD0901, previously known as CMG901, is an ADC with a humanized anti-CLDN18.2 antibody conjugated with monomethyl auristatin E (MMAE), a microtubule-disrupting agent, via cleavable linkers [27]. It has been shown to have direct MMAE-mediated cytotoxic effects on CLDN18.2-overexpressing tumor cells, as well as ADCC and CDC action and bystander-killing effects on surrounding tumor cells in preclinical studies [64,65,66]. Early data suggest that AZD0901 is well tolerated and has promising anti-tumor activity in CLDN18.2-positive tumors.

KYM901, a phase I trial, aimed to evaluate safety and anti-tumor activity of AZD0901 in patients with advanced solid tumors, including GC/GEJC and PC [53]. The dose-escalation phase enrolled 27 patients (13 [48%] with GC/GEJC and 14 [52%] with PC). The dose-expansion phase enrolled 107 patients with CLDN18.2-positive GC/GEJC. CLDN18.2-positivity was defined as membrane staining intensity of ≥2+ in ≥5% of tumor cells. In general, among the 113 patients with advanced GC/GEJC across both dose-escalation and dose-expansion phases included in the response analysis, the confirmed ORR was 28% (95% CI: 20–38) and the DCR was 63% (95% CI: 53–72). The median DoR was 7.9 months (95% CI: 5.3–10.0) [64]. The median PFS was 3.7 months (95% CI: 3.3–4.9) and the median OS was 10.1 months (95% CI: 6.8–13.9) at a median follow-up of nine months. Among the 32 (28%) patients who had a confirmed ORR across subgroups, 31 (97%) had CLDN18.2 membrane staining of at least 2+ intensity in at least 20% of tumor cells. This became their cutoff for what they consider CLDN18.2-high tumors. In total, 93 (82%) of the 113 patients with GC/GEJC fulfilled the criteria for CLDN18.2-high tumors. In these patients, the confirmed ORR was 33% (95% CI: 24–44), whereas in those with CLDN18.2 of at least 2+ intensity in less than 20% of tumor cells had an ORR of 5% (95% CI: 0–26). The patients with CLDN18.2-high tumors had improved mPFS of 4.8 months (95% CI: 3.4–6.1) and the mean OS was 11.8 months (95% CI: 7.0–14.7) at a median follow-up of 10.1 months [64].

The most commonly reported TEAEs included anemia (71%), hypoalbuminemia (61%), weight loss (59%), nausea (57%), vomiting (56%), decreased neutrophil count (53%), decreased white blood cell (WBC) count (51%), and decreased appetite (50%). Grade ≥ 3 TEAEs occurred in 68% of patients, most frequently decreased neutrophil count (21%), anemia (14%), and vomiting (10%). Low-grade peripheral neuropathy (grade 1–2) was observed in 20% of patients, consistent with the known neurotoxicity associated with MMAE-based ADCs, regardless of target antigen [64]. Data specific to the PC cohort will be reported separately.

The CLARITY-PanTumor01 study, an ongoing phase II trial with multiple substudies, is evaluating the use of AZD0901 in patients with CLDN18.2-positive advanced solid tumors, including GC/GEJC, PC, and BTCs [65]. Primary completion is estimated for December 2025. Substudy 1 will evaluate AZD0901 monotherapy in patients with CLDN18.2-positive, HER2-negative unresectable or metastatic GC/GEJC with ≤2 prior lines of therapy. Substudy 2 is recruiting patients with previously untreated CLDN18.2-positive advanced or metastatic PC, and patients will receive AZD0901 combined with chemotherapy. Substudy 3 will evaluate preliminary anti-tumor activity of AZD0901 monotherapy in patients with CLDN18.2-positive advanced or metastatic BTCs with ≤2 prior lines of therapy.

The GEMINI-Gastric study, a phase II, open-label, multi-drug, multi-center study, is looking to evaluate the efficacy, safety, tolerability, pharmacokinetics, and immunogenicity of multiple novel immuno-oncology (IO) agents plus chemotherapy combinations in patients with previously untreated HER2-negative advanced or metastatic GC/GEJC [67]. Three cohorts of the study include AZD0901 as part of the experimental combination regimens, along with chemotherapy (5-FU or capecitabine) and various novel BsAbs targeting immune checkpoints or immunomodulators.

There is an ongoing phase III trial (NCT06346392), known as the CLARITY-Gastric01 trial, which will evaluate the use of AZD0901 monotherapy vs. investigator-choice chemotherapy for patients with previously treated CLDN18.2-positive metastatic GC/GEJC [66].

#### 4.2.2. LM-302 (TPX4589/Tecotabart Vedotin)

LM-302, also known as TPX4589 or tecotabart vedotin, is another ADC targeting CLDN18.2 and conjugated to MMAE, the same cytotoxic payload as AZD0901. The safety and efficacy of LM-302 monotherapy is currently being investigated in a phase I/II trial for previously treated advanced or metastatic GC/GEJC (NCT05161390) [68]. In the dose-expansion phase, 52 patients with CLDN18.2-positive tumors (IHC ≥ 2+ in ≥50% of tumor cells) were enrolled. The ORR was 30.6% and the DCR was 75%. The mPFS was 7.16 months. The mean OS was not reached, with an OS rate of 95% at six months. The most common TRAEs observed were decreased WBC count (51.9%), decreased neutrophil count (51.5%), anemia (38.5%), vomiting (36.3%), and nausea (34.1%). The most frequent grade ≥ 3 TRAEs were decreased neutrophil count (22.2%) and decreased WBC count (17.8%) [68]. These findings suggest that LM-302 is generally well tolerated and shows promising anti-tumor activity in refractory CLDN18.2-positive GC/GEJC, supporting further investigation.

In a phase I/II study analysis, first-line treatment with LM-302 plus toripalimab (an anti-PD-1 antibody) for advanced HER2-negative GC/GEJC or EAC demonstrated promising efficacy and a manageable safety profile (NCT05188664, NCT05934331) [69]. A total of 43 patients were treated across various dosing regimens of LM-302 and toripalimab. Among 41 efficacy-evaluable patients (median follow-up: 6.01 months), the ORR was 65.9% (95% CI: 49.4–79.9%), and the DCR was 85.4% (95% CI: 70.8–94.4%). Notably, in a subgroup of 32 GC patients with moderate-to-high (IHC 2+/3+) CLDN18.2 expression in ≥25% of tumor cells, the ORR and DCR rose to 71.9% (95% CI: 53.3–86.3%) and 96.9% (95% CI: 83.3–99.9%), respectively. Efficacy was observed across PD-L1 expression levels. Patients with PD-L1 CPS <1 had an ORR of 63.3% (95% CI: 35.1–87.2%), while those with CPS ≥ 1 achieved an ORR of 77.8% (95% CI: 52.4–93.6%). Median PFS and OS were not reached at data cutoff. No dose-limiting toxicities (DLTs) were observed. TRAEs occurred in 90.7% of patients, including anemia, decreased neutrophil count, increased liver enzymes, vomiting, loss of appetite, and nausea. Grade ≥ 3 TRAEs were seen in 37.2% of patients, most commonly decreased neutrophil count (14.0%), elevated liver enzymes (ALT 11.6%, AST 9.3%), and anemia (7.0%). Overall, these encouraging findings support further evaluation of LM-302 plus toripalimab in larger, randomized trials targeting CLDN18.2-positive gastric, GEJ, and EAC, including those with low-to-moderate CLDN18.2 expression.

In a phase Ib/II, known as ZSAB-Calm, the efficacy and safety of LM-302 in combination with cardonilimumab (BsAb to PD-1 and CTLA-4) in patients with CLDN18.2-positive refractory advanced BTC [70]. Preliminary findings from the dose-escalation and dose-expansion phases showed a manageable safety profile with promising anti-tumor activity [70]. Among six evaluable patients, three achieved PR, yielding the best overall response (BOR) of 50% in phase Ib. No DLTs were observed. All patients in the dose-escalation cohort experienced TRAEs, with the most common being bilirubin increase (71.4%), thrombocytopenia (57.1%), neutropenia (57.1%), infusion reactions (rash) (42.9%), leukopenia (42.9%), and anemia (28.6%) [70].

#### 4.2.3. SHR-A1904 and IBI343

SHR-A1904 and IBI343 are both ADCs with similar compositions: an anti-CLDN18.2 mAb conjugated to a topoisomerase inhibitor payload in order to enact localized direct cytotoxic activity. These ADCs have a different mechanism of action than AZD0901 and LM-302, as they are loaded with topoisomerase inhibitors rather than microtubule inhibitors, so in the future they may offer an alternative to patients who have already received taxanes or failed ADCs with MMAE payloads. Phase I trial results for both SHR-A1904 and IBI343 in patients with advanced GC/GEJC were published on July 2025 [71,72].

Out of 88 patients that received SHR-A1904 and were evaluable for tumor response, the confirmed ORR was 19.3% (95% CI: 11.7–29.1); 52 (59.1%) patients had SD, and the DCR was 78.4% (95% CI: 68.4–86.5) [67]. The median PFS was 4.8 months (95% CI: 3.0–6.4), and 59 (62.1%) patients had disease progression or death as of the data cutoff. Among patients with moderate-to-high CLDN18.2 expression (≥2+ in ≥50% tumor cells), the confirmed ORR was 23.0% (95% CI: 14.0–34.2) [71].

The IBI343 trial showed equally promising efficacy results. Patients with high expression of CLDN18.2 (≥2+ in ≥75% tumor cells) GC/GEJC demonstrated an ORR of 29.0% (95% CI: 14.2–48.0) in the 6 mg/kg cohort (n = 31) and 47.1% (95% CI: 23.0–72.2) in the 8 mg/kg cohort (n = 17) [72]. The median PFS for each cohort was 5.5 months (95% CI: 4.1–7.0) and 6.8 months (95% CI: 2.8–7.5), respectively. In those with moderate-to-high CLDN18.2 (≥2+ in ≥40% tumor cells) that could be evaluated, the ORR was 38.8% (95% CI: 25.2–53.8) in the 6 mg/kg cohort (n = 49) and 44.8% (95% CI: 26.4–64.3) in the 8 mg/kg cohort (n = 29) [72].

The safety profile of both agents was similar. They primarily presented with hematologic (including anemia and decreased white blood cell count) and GI toxicities (including nausea and vomiting). The hematologic toxicities likely stem from the topoisomerase inhibitor payload, while the GI toxicities are likely both target- and payload-related [71,72]. No cases of interstitial lung disease (ILD), a commonly reported toxicity of currently approved first-generation ADCs, were noted in either trial to date. Nevertheless, given that the CLDN18.1 isoform is found in lung tissue, ongoing vigilance for pulmonary toxicity remains warranted.

Although currently only having phase I results, IBI343 and SHR-A1904 have shown promising efficacy and safety, leading to the development of phase II and phase III trials, as shown alongside other clinical trials in Table 2 below.

### 4.3. Bispecific Antibodies (BsAbs)

BsAbs are designed to bind both a tumor antigen (in this case, CLDN18.2) and immune effector cells or checkpoint molecules, thereby enhancing immune-mediated tumor cell killing [39]. Some of the drugs in this category that are further along in development are discussed below, and a list of ongoing clinical trials can be found in Table 3.

#### 4.3.1. Givastomig (TJ033721/ABL111)

Givastomig, also known as TJ033721 or ABL111, is a BsAb targeting CLDN18.2 and 4-1BB. 4-1BB, or CD137, is a co-stimulatory molecule from the TNF family that is normally expressed on antigen-presenting cells such as macrophages or B cells, and it contributes to activating CD8+ T-cells’ proliferation, survival, and cytotoxic effects [80]. Isolated agonistic 4-1BB mAbs have previously been explored as a potential immunotherapy approach for cancers, but were found to have significant hepatotoxicity [81]. Leveraging CLDN18.2’s specificity to gastric tissue and ectopic expression in malignancy, givastomig has presented a way to target the disease and minimize off-target effects. In fact, a preclinical mice study of givastomig found a strong anti-tumor effect without hepatotoxicity or cytokine release syndrome (CRS) when combining CLDN18.2 and 4-1BB targeting in GC/GEJC cells, paving the way for phase I trials [81].

There is an ongoing multi-center phase I study of givastomig in combination with nivolumab and chemotherapy (mFOLFOX) in patients with advanced or metastatic solid tumors (NCT04900818), with recently published results at the ESMO GI Cancers Congress 2025. CLDN18.2 positivity was defined as ≥1+ intensity in ≥ 1% of tumor cells. As of February 2025, 17 patients were treated across three dose levels of givastomig (5 mg/kg [n = 5], 8 mg/kg [n = 6], and 12 mg/kg [n = 6]). The combination was well tolerated, with no DLTs observed and a maximum tolerated dose (MTD) was not reached [75].

The most common TRAEs (≥10% of patients) included nausea (47%), vomiting (29%), infusion-related reactions (29%), and fatigue (24%). Grade 3 TRAEs were rare and included isolated cases of abdominal pain, elevated liver enzymes (ALT/AST), gastritis, and infusion-related reactions. No grade 4 or 5 TRAEs were observed. Pharmacokinetics showed dose-dependence and aligned with prior monotherapy profiles [75].

Givastomig demonstrated robust anti-tumor activity with an ORR of 71% (12/17 patients), including responses across all dose levels. In the expansion-dose cohorts (8 and 12 mg/kg), ORR reached 83%. The DCR was 100%. Responses were observed regardless of PD-L1 CPS (CPS ≥ 5 or <5) or CLDN18.2 expression levels (≥75% or <75%). At the time of data cutoff, eight patients remained on treatment, with the longest duration being 11.3 months [75]. These preliminary results suggest that givastomig in combination with nivolumab and chemotherapy is both safe and highly active as first-line therapy for metastatic GC/GEJC and EACs, with dose expansion ongoing. Particularly, these results show that responses to givastomig were seen in patients with low CLDN18.2 expression levels who would not normally be eligible for CLDN18.2-targeted treatments.

#### 4.3.2. ASP2138

ASP2138 is an IgG-based BsAb targeting CLDN18.2 and the T-cell-associated CD3 antigen to facilitate cytotoxicity in the TME. Preclinical trials showed promise in mice cancer models with increased cytotoxicity observed in CLDN18.2-expressing tumor cells, as well as increased expression of interferon-gamma and T-cell activation markers [82]. There is an ongoing phase I clinical trial for patients with CLDN18.2-positive GC/GEJC and PC (NCT05365581).

#### 4.3.3. QLS31905

QLS31905 is another CLDN18.2/CD3 BsAb that has recently published phase I safety and efficacy data [77] involving patients with progressive or inoperable solid tumors, irrespective of CLDN18.2 status. The study included 31 patients in the dose-escalation stage and 48 patients with CLDN18.2-positive tumors (defined as ≥1% tumor cells on IHC) in the dose-expansion stage. The agent was overall well tolerated, with no DLTs recorded and the MTD was not reached during the dose-escalation stage. TRAEs occurred in 79 (100%) patients and 43.04% were grade ≥ 3. The most common grade ≥ 3 TRAEs were lymphocyte count decrease (21.5%), GGT increase (3.8%), neutrophil count decrease (3.8%), CRS (3.8%), and anemia (3.8%).

Among 33 patients with CLDN18.2-positive GC/GEJC and PC receiving 350–1200 μg/kg q2w, six patients had a PR (three with GC/GEJC and three with PC) and the ORR was 18.18% (95% CI: 6.98–35.46%). The DCR was 87.88% (95% CI: 71.80–96.60%), the median PFS was 4.21 months (95% CI: 2.99–5.55), and the median OS was 9.53 months (95% CI: 7.69-NE). In 19 patients with CLDN18.2-positive GC/GEJC, the ORR was 15.79%, the DCR was 89.47%, the median PFS was 4.40 months, and the median OS was 9.20 months. In 12 patients with CLDN18.2-positive PC, the ORR was 25.00%, the DCR was 91.67%, the median PFS was 3.94 months, and the median OS was not reached.

These early data suggest that QLS31905 has encouraging activity against both GC/GEJC and PC with tolerable safety profile, and phase II-III trials are underway to further evaluate its efficacy. This includes phase II trials employing QLS31905 in combination with chemotherapy (NCT06041035, NCT06446388) and QL2107, a newly developed agent that is biosimilar to pembrolizumab (NCT06942767). There is also a phase III trial planned to start in September 2025 evaluating the efficacy of QLS31905 in combination with chemotherapy (nab-paclitaxel plus gemcitabine) against placebo for CLDN18.2-positive advanced PC (NCT07079228). To our knowledge, this is the first planned phase III trial for a CLDN18.2-directed agent specifically for PC.

### 4.4. CAR T-Cell Therapy

CAR T-cell therapy involves genetically modifying cytotoxic T-cells to have a CAR that recognizes specific tumor antigens [16]. CARs are typically added via a lentiviral vector that is introduced to a patient’s own CD8+ T-cells that have been extracted [83]. These CAR T-cells then proliferate ex vivo until they are re-infused into the patient with malignancy, often after receiving lymphodepleting chemotherapy [83]. Once in the body, the CAR T-cells’ co-stimulatory domains bind to the target antigen and enact their cytotoxic effect. This advancement has revolutionized the treatment of certain hematologic malignancies, and recent research has examined their efficacy in solid malignancies including GC and PC. While many agents are still in early-phase trials, as shown in Table 4, this section highlights some of the agents that are at more advanced stages of development.

#### 4.4.1. CT041/Satri-cel

CT041/Satri-cel is still in early phase I/II trials. A phase I trial (NCT03874897) that included 98 patients with CLDN18.2-positive advanced GI cancers found an ORR and DCR of 57.4% and 83.0%, respectively, in evaluable GC patients with CT041 monotherapy. The median PFS and median OS were 5.8 months (95% CI 4.2–8.4) and 9.7 months (95% CI 7.1–14.4), respectively [89]. The most common TEAEs of grade ≥ 3 were hematologic toxicity related to lymphodepletion. CRS occurred in 96.9% of patients, all were grade 1–2. No DLT was observed [89]. This study showed an encouraging efficacy and safety profile in heavily pretreated patients with CLDN18.2-advanced GI cancers. Table 4 shows updated overall efficacy data for CT041 as monotherapy and in combination with anti-PD-1 therapy in CLDN18.2-positive advanced GI cancers [85].

Satri-cel has also been evaluated in PC, where it showed an encouraging efficacy and safety profile in patients with CLDN18.2-positive previously treated metastatic PC [87]. The ORR was 16.7% and the DCR was 70.8%. The median PFS was 3.3 months (95% CI: 1.8–6.2) and the median OS was 10.0 months (95% CI: 5.5–17.6) [87]. The median DoR was 9.5 months (95% CI: 2.6-NE) [87]. The safety profile was similar to the study above.

In the phase Ib/II ELIMYN18.2 trial, notable clinical activity was also observed in heavily pretreated patients with CLDN18.2-positive GC/GEJC and PC [86]. Patients were first conditioned with chemotherapy, followed by 1–3 cycles of satri-cel. Across the treated population, the best ORR reached 26.3%, with a median DoR of 3.7 months and a CBR of 42.1% [86].

More recently, the results of CT041-ST-01, the first confirmatory randomized control trial for CAR T-cell therapies in solid tumors globally, were published in May 2025. This pivotal phase II trial evaluated satri-cel against treatment of physician’s choice (TPC) as third-line treatment in advanced GC/GEJC with CLDN18.2 positivity (defined as ≥2+ in ≥40% tumor cells on IHC) [90]. A total of 156 patients were randomly assigned to either satri-cel (n = 104) or TPC (n = 52). Participants in the TPC arm who experienced disease progression or could not tolerate the drug could receive subsequent satri-cel treatment if eligible. Twenty patients in the TPC arm received subsequent satri-cel. In the intention-to-treat population, the satri-cel arm showed a significant improvement in median PFS compared with the TPC arm (3.25 months vs. 1.77 months; HR 0.366, 95% CI 0.241–0.557; *p* < 0.0001) [90]. The satri-cel arm also showed a longer median OS than the TPC arm (7.92 months vs. 5.49 months; HR 0.693; 95% CI: 0.457–1.051; one-sided *p* = 0.0416). Notably, the median OS for TPC patients who later received satri-cel was 9.20 months [90]. Among all patients who received satri-cel (n = 108) compared with TPC patients who did not (n = 28), median OS was 9.17 months versus 3.98 months (HR 0.288; 95% CI 0.169–0.492) [90]. Regarding safety, the most common grade ≥ 3 TEAEs included decreased lymphocyte count (98%), decreased white blood cell count (77%), and decreased neutrophil count (66%) [91]. CRS occurred in 95% of patients receiving satri-cel [91]. Overall, satri-cel demonstrated meaningful clinical benefits with improvements in PFS and OS and a manageable safety profile in previously treated CLDN18.2-positive GC/GEJC.

#### 4.4.2. TAC01-CLDN18.2

TAC01-CLDN18.2 is an agent consisting of genetically engineered autologous T-cells expressing a T-cell Antigen Coupler (TAC), which binds a target antigen and brings it to the cell’s natural T-cell receptor (TCR). This may induce both cytotoxic activity and cytokine release for further immune activation in a controlled manner, potentially making the treatment effect more long-lasting than traditional CAR T-cells [92]. Preclinical models of TAC01-CLDN18.2 showed highly CLDN18.2-specific cytotoxicity in GC and PC mice tumor xenografts with minimal nonmalignant cell effects [92]. TACTIC-3 is an ongoing phase I/II trial evaluating the safety and efficacy of this agent in CLDN18.2+/HER2- advanced solid tumors. Preliminary results have shown minimal adverse effects with evidence of clinical activity, but further evaluation is warranted [88].

Table 5 below shows a comparative analysis of the side-effect profiles of the different types of agents targeting CLDN18.2 just discussed, along with suggested mitigation strategies.

## 5. Co-Expression Patterns and Combination Therapies in Gastric/Gastroesophageal Junction Cancer

### 5.1. CLDN18.2 and PD-L1

Given the immunosuppressive TME of many GI malignancies, one of the future directions of CLDN18.2 research is combination therapies with ICIs. In general, PD-L1 expression is considered positive at a CPS cutoff of ≥1. The GLOW trial found co-existing CLDN18.2 positivity and PD-L1 CPS ≥ 5 in 21.9% (63/228) of patients [9]. The SPOTLIGHT trial showed CLDN18.2 and PD-L1 CPS ≥ 5 positivity in 13% (41/311) of patients [8]. When looking at the lower cutoff of CPS ≥ 1, a retrospective analysis showed that 74.2% of patients had both CLDN18.2 and PD-L1 positivity [22]. Another retrospective analysis showed consistent results, finding that 78.6% of patients with GC showed CLDN18.2 positivity (≥40%) as well as PD-L1 CPS ≥ 1 [93]. These findings suggest accordance rates up to roughly 80% of patients showing CLDN18.2 positivity and CPS ≥ 1 [94], which is of great clinical significance as a large amount of patients may potentially benefit from combination therapy.

Mechanistically, combination therapy with CLDN18.2-targeting drugs and ICIs has the potential of having a synergistic anti-tumor effect. mAbs aimed at CLDN18.2 depend on the recruitment of immune effector cells and bringing them into proximity to tumor cells to undergo ADCC and CDC. With the addition of ICIs, immune cells are subsequently more likely to recognize malignant cells for destruction and increase the antitumoral response. Early trials of ASKB589 suggest this effect, where the ORR was higher when combining CAPOX plus a PD-1 inhibitor than when used as monotherapy [55,63]. ADCs, particularly with microtubule inhibitor or topoisomerase inhibitor payloads, have also been proposed to have a combinatorial effect with ICIs by inducing immunogenic cell death [95]. When tumor cells are damaged by ADC-mediated cytotoxicity, they may release damage-associated molecular patterns (DAMPs), or molecules that can activate surrounding immune cells to cause an inflammatory response. By reducing immunosuppression, ICIs can mediate or facilitate this response [95].

The third arm of ILUSTRO, a phase II trial, included a cohort of zolbetuximab plus pembrolizumab in patients with recurrent locally advanced or metastatic CLDN18.2-positive GC/GEJC (NCT03505320). Preliminary efficacy findings in 2023 showed minimal benefit with no partial responses, though the sample size was small with three enrolled subjects [51]. However, there were no serious adverse effects in this population. Given that the sample size was too small to draw any conclusions, cohort 4 is currently enrolling patients to evaluate zolbetuximab in combination with FOLFOX and nivolumab as first-line therapy in patients with intermediate-to-high CLDN18.2-positive HER2-negative metastatic GC/GEJC. Moreover, a new phase III trial is underway evaluating chemotherapy (CAPOX or mFOLFOX6) and pembrolizumab, with or without zolbetuximab, as first-line therapy in advanced or metastatic GC/GEJC whose tumors are HER2-negative, CLDN18.2-positive, and PD-L1-positive (NCT06901531).

### 5.2. CLDN18.2 and HER2

To date, there are limited data evaluating the relationship between CLDN18.2 and HER2 expression in GI malignancies [94,96]. While some studies have estimated co-expression rates of 12–21%, most have not been statistically significant [21,93,97,98].

Currently, the FDA-approved use of zolbetuximab is limited to patients with HER2-negative GC/GEJC, and to our knowledge, there are no clinical trials targeting tumors with CLDN18.2 and HER2 co-expression. However, new agents are in development that have shown promise in preclinical studies. For instance, HC-2G4S is an anti-HER2 and CLDN18.2 BsAb that has shown encouraging in vitro anti-tumor potency in CLDN18.2+/HER2+ GC [99]. While there is not yet a clinical trial evaluating the safety of this agent, the development of BsAbs may lead to further avenues of multi-antigen targeting that could help avoid drug resistance in the future.

## 6. Possible Resistance Mechanisms to Anti-CLDN18.2 Therapy

Despite encouraging clinical results, possible resistance mechanisms must be anticipated, together with strategies to overcome them, as seen with prior targeted therapies. Resistance mechanisms include downregulation of CLDN18.2 expression, modulation of the TME, and impaired drug delivery.

### 6.1. Downregulation of CLDN18.2 Expression

One of the main resistance mechanisms of concern with CLDN18.2-targeted therapies is the downregulation or loss of CLDN18.2 expression. This has been seen previously in HER2-positive GC, where the loss of HER2 antigen expression has been found to develop in 69% of patients receiving HER2-directed therapy [100]. To our current knowledge, there are no direct data on CLDN18.2 expression changes after zolbetuximab or other CLDN18.2-directed treatment; however, loss of CLDN18.2 expression has been noted in 25–40% of GC patients after first-line chemotherapy [8,21,22,101].

The mechanisms behind antigen loss after antibody-mediated immunotherapy are variable and have been particularly studied in the setting of hematologic malignancies [102]. Malignant cells may select toward internalization of target antigens or have disrupted protein trafficking, thereby hiding them from the cell surface where an antibody-driven therapy may find it. Similarly, some hematologic antigens have been “shaved” off the cell surface via trogocytosis, mediated by immune phagocytic cells [102]. Although these mechanisms have not been researched specifically for CLDN18.2, they can inform future directions in addressing antigen loss as treatment resistance arises.

In PC cells, CLDN18.2 expression is downregulated at the transcriptional level by methylation of its promoter region [103]. Cytotoxic stress from first-line chemotherapy may increase DNA methylation and therefore decrease gene expression. Further research is needed to develop avenues for overcoming this resistance, and to identify the rates of antigen loss after CLDN18.2-directed therapies and its clinical impact.

One of the primary methods that has been employed to address antigen loss and as an avenue of resistance has been the development of BsAbs or the employment of combination therapies. As discussed above, there are multiple BsAbs in development that crosslink CLDN18.2 with immune-activating antigens such as CD3 or 4-1BB, which may upregulate the cytotoxic immune response such that it can overcome antigen loss and reach cells that are not expressing CLDN18.2 [81,82]. In addition, although zolbetuximab is currently approved only for use with chemotherapy in patients with HER2-negative advanced GC, combination strategies pairing zolbetuximab with immunotherapies are in development and may offer a promising approach to overcoming early treatment resistance.

### 6.2. Tumor Microenvironment (TME) Modulation

Another way in which tumors may become resistant to CLDN18.2-targeting drugs is by altering the TME. The TME is an important independent factor that plays a role in tumor progression, metastasis, and prognostication, particularly in patients with GC [104]. It is a complex system composed of a variety of cell types and noncellular substances that actively engage with malignant cells and plays a key role in cancer cell survival and invasion [105]. Depending on the type of cancer, this may include immune cells that can either promote or suppress tumor growth. In addition, stromal cells such as fibroblasts or endothelial cells interact with cancer cells in the TME to stimulate angiogenesis or cell motility. Given that the leading treatments targeting CLDN18.2 depend on ADCC and CDC to exert their antitumoral effect, their efficacy are likely significantly affected by the TME and the cellular interactions within [106].

A recent prospective study of GC biopsies examined the genetic makeup of the TME before and after chemotherapy and found that CLDN18.2-positive tumors were associated with more immune cell infiltration and stromal cell-mediated fibrosis [101]. Despite higher presence of immune cells, these cancers demonstrated activation of immunosuppressive pathways involving CD44 and galectin-3 that are frequently seen in ICI-resistant cancers. Therefore, these dynamic TMEs can develop resistance to ADCC- and CDC-dependent therapies like zolbetuximab. For instance, one study by Tsutsumi and colleagues examined the microenvironments of GC samples and found that CLDN18.2-positive samples treated with neoadjuvant chemotherapy were associated with lower expression of ADCC-related genes [107]. More research is needed to discern the efficacy of therapies like zolbetuximab earlier in the disease course and to identify the exact mechanistic pathways that contribute to this immune evasion.

One of the more promising strategies to address the TME is combination therapy with CLDN18.2 antibody targeting and ICIs. While zolbetuximab does not yet have data showing improved outcomes in combination with PD-1/PD-L1 inhibitors, trials are currently underway. Similarly, osemitamab is a new mAb that is currently undergoing phase II/III clinical trials to assess combinations with checkpoint inhibitors, as mentioned above.

Furthermore, the role of TGF-β and other cytokines in affecting the immune microenvironment has previously been described in the setting of PC and other GI cancers [108,109,110]. One of its signaling pathways can have a significant inhibitory effect on the function of cytotoxic T-cells, contributing to possible resistance to ADCC. The suppressive effect of TGF-β in the TME has also been noted to be a key obstacle in the efficacy of CAR T-cell therapies in GI tumors [111]. As such, TGF-β has become one possible target in developing combination therapies seen in current clinical trials. For example, AZD6422 is a CLDN18.2-targeted CAR T-cell therapy in development that has shown improved efficacy when augmented with armoring against TGF-β in preclinical testing [112]. Similarly, some preclinical studies are exploring CAR T-cells that co-target IL-10, another cytokine implicated in the immunosuppressive TME, or cancer-associated fibroblasts that normally suppress anti-tumor effects [113]. Given that immunosuppressive TMEs in solid tumors have been known to limit durable CAR T-cell responses, ICIs may synergistically reactivate exhausted CAR T-cells and are being evaluated in other cancer types [114].

Finally, one possibility of addressing the immunosuppressive TME is to explore new directly cytotoxic therapies with ADCs. One promising option that has been explored above is AZD0901, an ADC that targets CLDN18.2 to deliver the microtubule-disrupting agent MMAE, thereby directly killing tumor cells without depending on immune system activation. Similarly, two ADCs with recently published promising phase I data, IBI343 and SHR-A1904, add a cytotoxic topoisomerase I inhibitor payload to CLDN18.2 mAbs [71,72]. These may provide an approach to overcoming the immunosuppressive TME while potentially minimizing the adverse effects of systemic therapy. Newer preclinical studies are also exploring radionucleotide therapy with ADCs, where zolbetuximab is being loaded with radioactive iodine for a direct cytotoxic effect on the tumor [115].

### 6.3. Poor Drug Delivery and Tumor Infiltration

Besides frequently creating an immunosuppressive effect, the TME of GC and other GI malignancies can hamper adequate drug delivery. Through the recruitment of a variety of macrophages, fibroblasts, and other stromal cells, malignancies often develop dysfunctional vasculature and have a dense extracellular matrix that limits mAb delivery into tumors [116]. This impaired bioavailability in malignant tissue has been noted as a particular barrier to the development of CAR T-cell therapies that target CLDN18.2 [117].

The migration of CAR T-cells from the bloodstream to solid tumors depends on chemokines such as ligand-11 and 12 or ICAM-1, which are often downregulated in GI malignancies and help explain their limited effectiveness in solid tumors compared to hematologic malignancies [111]. Additionally, CAR T-cells are suppressed or rendered dysfunctional by the TME’s lack of nutrients, low pH, lactate, and other metabolites [117].

To overcome this challenge, future research will explore improving CAR T-cell delivery to solid tumors. One example in early development is the employment of small extracellular vesicles (sEVs). These are naturally secreted particles that are believed to offer improved delivery of cytotoxic T-cells or immune modulators by avoiding the barriers of the TME that prevent entry of traditional CAR T-cells, mAbs, or other therapeutic agents. In a recent study of CAR T-cells delivered with sEVs in PC mice models, treatment with CAR-sEVs targeted at CLDN18.2 showed significantly prolonged survival and reduced tumor burden compared to controls [118].

Collectively, these resistance mechanisms highlight the importance of developing multi-targeted approaches, optimizing combinations, and tailoring treatment based on tumor biology.

## 7. Conclusions

CLDN18.2 has emerged as one of the more promising therapeutic targets for GI cancers, particularly GC. Anti-CLDN18.2 therapies, especially mAbs such as zolbetuximab, have shown substantial promise in clinical trials for the treatment of CLDN18.2-positive tumors. Clinical trials like SPOTLIGHT and GLOW have demonstrated that targeting CLDN18.2 can significantly improve PFS and OS in patients with advanced GC/GEJC whose tumors express high levels of CLDN18.2 [8,9]. These results validate CLDN18.2 as a clinically actionable biomarker and support its incorporation into treatment algorithms. Similarly, recent encouraging preclinical and early-phase data are introducing alternate drug classes with distinct mechanisms of actions and adverse effect profiles as potential avenues for CLDN18.2-positive GI tumors. BsAbs, ADCs, and CAR T-cell therapy present unique avenues to target GI cancers from varied and potentially synergistic mechanisms of action.

This narrative review uniquely synthesizes the expanding landscape of CLDN18.2-directed therapies across gastric, pancreatic, and biliary tract cancers, moving beyond zolbetuximab to highlight diverse next-generation modalities, combination immunotherapy approaches, and emerging engineering platforms. By comparing safety profiles, identifying toxicity mitigation strategies, examining CLDN18.2/PD-L1 co-expression, and evaluating mechanisms of resistance with proposed solutions, we offer a comprehensive framework to guide clinical translation and research innovation.

### Future Directions

Despite current advances in the field, several challenges remain that affect clinical implementation. Future research directions should prioritize the following:

(1) How to integrate CLDN18.2 targeted therapies with ICIs in GC/GEJC, which have become standard of care. This is important given the observed co-expression of CLDN18.2 and PD-L1 in subsets of patients [94]. Prospective clinical trials evaluating the safety and efficacy of combination regimens are needed to guide sequencing and optimize outcomes.
(2) Identifying which levels of CLDN18.2 expression stand to benefit most from different targeting strategies. While zolbetuximab requires high expression (≥75% of tumor cells with 2+/3+ membranous staining), other agents may prove effective at intermediate or even low expression levels, as suggested with agents such as AZD901 and LM-302. This opens the door to tailoring therapeutic strategies based on expression intensity, potentially expanding eligibility to broader patient populations. Standardization of CLDN18.2 testing will be essential to identify appropriate candidates for treatment.
(3) Developing novel approaches such as ADCs and BsAbs targeting CLDN18.2 to overcome resistance. Enhanced efficacy through payload delivery or dual-targeting strategies may overcome mechanisms of resistance including antigen loss, tumor heterogeneity, and immune evasion [39].
(4) Expanding the application of CLDN18.2-targeted therapies to other GI malignancies, including PC and BTCs. Approximately one-third of PCs and a smaller subset of GBCs and eCCAs have been shown to express CLDN18.2 at therapeutically actionable levels [34,35,36]. This review has highlighted ongoing and emerging clinical trials that evaluate the efficacy of CLDN18.2-targeted agents in these cancers, which historically lack effective targeted treatments. The adoption of standardized testing across tumor types, accounting for intratumoral heterogeneity and primary–metastatic discordance, will be essential to identify potential patients eligible for clinical trials targeting CLDN18.2.

In summary, CLDN18.2-targeted therapies represent a major advance in the management of select GI cancers. Focusing future efforts in the above domains will enable us to refine which patient populations would best benefit from the expanding variety of CLDN18.2-targeted therapy approaches in precision oncology.

## Figures and Tables

**Table 1:** Monoclonal antibodies (mAbs) targeting CLDN18.2 in trials.

Agent	NCT	Phase	Enrollment	Subjects	Combination(s)	Outcomes ^†^	Status
Zolbetuximab	NCT01197885	II	54	GC/GEJC	monotherapy	ORR: 9% [50]	Completed
Zolbetuximab	NCT01630083	II	252	GC/GEJC, esophageal	+ chemotherapy	PFS: 7.5 mo, HR (0.44, 95% CI 0.29–0.67) [23]	Completed
Zolbetuximab	NCT03505320	II	143	GC/GEJC	+/− chemotherapy or ICI	monotherapy: ORR 0% + Chemo: ORR 71.4%, mPFS 17.8 mo + ICI: ORR 0% [51]	Active, not recruiting
Zolbetuximab	NCT03816163	II	393	PC	+ chemotherapy	---	Active, not recruiting
Zolbetuximab	NCT03653507	III	507	GC/GEJC	+ chemotherapy	PFS 8.2 mo (HR 0.771) [9]	Active, not recruiting
Zolbetuximab	NCT03504397	III	565	GC/GEJC	+ chemotherapy	PFS 10.6 mo (HR 0.75) [8]	Active, not recruiting
Zolbetuximab	NCT06901531	III	500 *	GC/GEJC	+ chemotherapy + ICI	---	Recruiting
TST001	NCT04396821	I/II	150 *	solid tumors	+/− chemotherapy +/− ICI	---	Active, not recruiting
TST001	NCT04495296	I/II	320 *	solid tumors	+/− chemotherapy +/− ICI	+ Chemo: ORR 65.4% [52] + Chemo + ICI: ORR 55.7% [53]	Recruiting
TST001	NCT05190575	II	8	BTC	monotherapy	---	Completed
TST001	NCT06093425	III	950 *	GC/GEJC	+ chemotherapy + ICI	---	Not yet recruiting
ASKB589	NCT04632108	I/II	214 *	solid tumors	+ chemotherapy	ORR 31.1%, DCR 71.4% [54]	Recruiting
ASKB589	NCT05632939	I/II	57 *	GC/GEJC	+ chemotherapy + ICI	ORR 73.5% [55]	Recruiting
ASKB589	NCT06206733	III	780 *	GC/GEJC	+ chemotherapy + ICI	---	Recruiting
FG-M108	NCT04894825	I/II	152 *	solid tumors	+ chemotherapy	ORR: 78% in GC [56], ORR: 53% in PC [57]	Recruiting
FG-M108	NCT06177041	III	486 *	GC/GEJC	+ chemotherapy	---	Recruiting

* = estimated. ^†^ preliminary efficacy data included when available. Information obtained from clinicaltrials.gov.

**Table 2:** Antibody–drug conjugates (ADCs) targeting CLDN18.2 in trials.

Agent	NCT	Phase	Enrollment	Subjects	Combination(s)	Outcomes ^†^	Status
AZD0901	NCT04805307	I	176	solid tumors	monotherapy	ORR 28%, DCR 63%, mPFS 3.7 mo [64]	Completed
AZD0901	NCT06219941	II	190 *	solid tumors	+/− chemotherapy	---	Recruiting
AZD0901	NCT05702229	II	240 *	GC/GEJC	+ chemotherapy + ICI	---	Recruiting
AZD0901	NCT06346392	III	572 *	GC/GEJC	monotherapy	---	Recruiting
LM-302	NCT05994001	I/II	96 *	BTC	+ ICI	---	Recruiting
LM-302	NCT05161390	I/II	206 *	solid tumors	monotherapy	ORR 30.6%, DCR 75% [68]	Active, not recruiting
LM-302	NCT05934331, NCT05188664	I/II	276 *	solid tumors	+ ICI	ORR 65.9%, DCR 85.4% [69]	Recruiting
LM-302	NCT06587425	II	50 *	GC/GEJC	+ chemotherapy + ICI	---	Recruiting
LM-302	NCT06351020	III	375 *	GC/GEJC	monotherapy	---	Recruiting
SHR-A1904	NCT04877717	I	107	solid tumors	monotherapy	ORR 24.2% [71]	Active, not recruiting
SHR-A1904	NCT06350006	I/III	924 *	solid tumors	+ chemotherapy + ICI	---	Recruiting
SHR-A1904	NCT06649292	III	524 *	GC/GEJC	monotherapy	---	Recruiting
IBI343	NCT05458219	I	127	GC/GEJC	monotherapy	ORR 29%, mPFS 5.5 mo [72]	Recruiting
IBI343	NCT07025889	I/II	55 *	GC/GEJC	+ ICI	---	Recruiting
IBI343	NCT06770439	II	64 *	PC	+ chemotherapy	---	Not yet recruiting
IBI343	NCT06238843	III	450 *	GC/GEJC	monotherapy	---	Enroll by invitation
IBI343	NCT07066098	III	201 *	PC	monotherapy	---	Not yet recruiting
RC118	NCT05205850	I/II	135 *	solid tumors	monotherapy	ORR 47.1% [73]	Recruiting
TQB2103	NCT05867563	I	71 *	solid tumors	monotherapy	ORR 20%, DCR 76.7% [74]	Unknown status
ATG-022	NCT05718895	I	156 *	solid tumors	monotherapy	---	Recruiting

* = estimated. ^†^ preliminary efficacy data included when available. Information obtained from clinicaltrials.gov.

**Table 3:** Bispecific antibodies (BsAbs) targeting CLDN18.2 in trials.

Agent	NCT	Phase	Enrollment	Subjects	Combination(s)	Outcomes ^†^	Status
Givastomig	NCT04900818	I	168 *	GC/GEJC, EAC	+ chemotherapy + ICI	ORR 71% [75]	Recruiting
ASP2138	NCT05365581	I	378 *	GC/GEJC, PC	monotherapy	DCR 41.7% [76]	Recruiting
QLS31905	NCT05278832	I	104 *	solid tumors	monotherapy	ORR 18.2%, DCR 87.9% [77]	Unknown status
QLS31905	NCT06041035	I/II	115 *	solid tumors	+ chemotherapy	---	Not yet recruiting
QLS31905	NCT07079228	III	602 *	PC	+ chemotherapy	---	Not yet recruiting
Q-1802	NCT04856150	I	66 *	solid tumors	monotherapy	---	Unknown status
Q-1802	NCT05964543	I/II	72 *	GC/GEJC	+ chemotherapy	---	Recruiting
SG1906	NCT05857332	I	60 *	solid tumors	monotherapy	---	Recruiting
PT886	NCT05482893	I/II	258 *	GC/GEJC, PC, BTC	+/− chemotherapy +/− ICI	---	Recruiting
AZD5863	NCT06005493	I/II	240 *	solid tumors	monotherapy	---	Recruiting
PM1032	NCT05839106	I/II	200 *	solid tumors	monotherapy	ORR 20% [78]	Recruiting
LB4330	NCT06468358	I/II	194 *	solid tumors	monotherapy	---	Recruiting
IBI389	NCT05164458	I	320 *	solid tumors	monotherapy	ORR 30.8%, DCR 73.1% [79]	Recruiting

* = estimated. ^†^ preliminary efficacy data included when available. Information obtained from clinicaltrials.gov.

**Table 4:** CAR T-cell therapy targeting CLDN18.2 in trials.

Agent Name	NCT	Phase	Enrollment	Subjects	Combination(s)	Outcomes ^†^	Study Status
CT041	NCT03159819	I	12	GC, PC	monotherapy	ORR 33.3%, mPFS 130 days [84]	Unknown status
CT041	NCT03874897	I	98	solid tumors	monotherapy +/- ICI	ORR 38.8%, DCR 91.8%, mPFS 4.4 mo, mOS 8.8 mo [85]	Completed
CT041	NCT04404595	I/II	110 *	GC/GEJC, PC	monotherapy	ORR 26.3%, DOR 3.7 mo, mPFS 3.3 mo, mOS 8.9 mo [86]	Active, not recruiting
CT041	NCT04581473 NCT03874897	I/II	192 *	PC	monotherapy	ORR 16.7%, DCR 70.8%, mPFS 3.3 mo, mOS 10.0 mo [87]	Active, not recruiting
IMC002	NCT05472857	I	30 *	solid tumors	monotherapy	---	Recruiting
IMC002	NCT05946226	I	18 *	GI tumors	monotherapy	---	Recruiting
IMC008	NCT05837299	I	18 *	solid tumors	monotherapy	---	Recruiting
KD-496	NCT05583201	I	18 *	solid tumors	monotherapy	---	Recruiting
IBI345	NCT05199519	I	7	solid tumors	monotherapy	---	Completed
LB1908	NCT05539430	I	56 *	GC/GEJC, PC	monotherapy	---	Recruiting
TAC01-CLDN18.2	NCT05862324	I/II	113 *	solid tumors	monotherapy	DCR 100% [88]	Active, not recruiting
AZD6422	NCT05981235	I	8	solid tumors	monotherapy	---	Completed
CT048	NCT05393986	I	63 *	solid tumors	monotherapy	---	Unknown status

* = estimated. ^†^ preliminary efficacy data included when available. Information obtained from clinicaltrials.gov.

**Table 5:** Safety profile comparison among the different therapeutic modalities.

**Modalities**	Most Common TRAEs	Mechanistic Rationale	Mitigation Strategies
mAbs	Nausea, vomiting, decreased appetite	On-target binding in gastric mucosa (CLDN18.2 in normal mucosa)	Prophylactic antiemetics; slow infusion rate; hold/reduce dose per protocol
BsAbs	Nausea and vomiting; infusion reactions (less common)	Similar on-target gastric effects; potential T-cell engagement component	Antiemetics; premedication for infusion reactions; infusion rate control
ADCs	Hematologic AEs; GI AEs (generally lower grade vs. mAbs)	Payload-mediated marrow toxicity; some on-target/off-tumor effects	CBC monitoring; infection risk reduction; antiemetics; infusion adjustments
CAR T	CRS (grade 1–2); hematologic cytopenias (very common)	Immune activation and lymphodepletion; less on-target gastric effects	CRS support +/− steroids, tocilizumab; antimicrobial prophylaxis; CBC monitoring

## Data Availability

No new data were created or analyzed in this study.
